# Phthalate-induced effects in algae and fishes: insights into environmental toxicology

**DOI:** 10.1007/s11356-026-37581-7

**Published:** 2026-03-10

**Authors:** Dario Savoca, Chiara Martino, Antonella Maccotta, Vincenzo Arizza, Manuela Mauro, Mirella Vazzana, Alessandro Vaglica

**Affiliations:** 1 Department of Biological, Chemical and Pharmaceutical Sciences and Technologies (STEBICEF), University of Palermo, 90100 Palermo, Italy; 2 NBFC, National Biodiversity Future Center, Palermo, 90133 Italy

**Keywords:** Phthalic acid esters, Endocrine-active substances, Aquatic pollution, Physiological stress responses, Ecotoxicology, Ecosystem health, Life stage vulnerability

## Abstract

Phthalic acid esters (PAEs) are ubiquitous environmental contaminants with endocrine-disrupting properties and increasing relevance for aquatic ecosystem health. This review synthesizes current evidence on PAEs’ effects in fishes and micro- and macroalgae, integrating molecular, physiological and ecological responses across key trophic levels. In algae, PAEs impair photosynthesis, growth and metabolic homeostasis, while revealing a dual role as both targets of toxicity and potential contributors to environmental phthalate pools. In fishes, PAEs disrupt endocrine regulation, reproduction, development, metabolism and neurobehaviour, with emerging evidence of cardiotoxicity and epigenetic effects. By jointly analysing primary producers and vertebrate consumers, this review highlights species- and life-stage-specific vulnerabilities under environmentally realistic exposure scenarios, including chronic low-dose contamination, metabolite toxicity and mixture effects. Oxidative stress and endocrine signalling emerge as convergent mechanistic pathways. The review also evaluates algae-based bioremediation strategies, emphasizing species-dependent efficiencies and mixture-driven limitations. Major knowledge gaps include scarce long-term and multi-stressor studies and limited understanding of cross-trophic mechanisms. Overall, this integrated synthesis supports improved ecological risk assessment, informs regulatory frameworks and guides future multi-trophic and mechanistically driven research to mitigate PAEs pollution.

## Introduction

Phthalates, or phthalic acid esters (PAEs), are a group of chemicals frequently used to enhance flexibility and durability in a wide variety of consumer products like polyvinyl chloride (PVC), construction materials, pesticides, packaging, medicines, personal care products and toys (Pham et al. 2025). The worldwide production of PAEs is presently estimated at 300 million tons, which can be approximated to be 500 million tons in the year 2050, and most of them will be utilized once (Huang et al. 2021).

PAEs are ubiquitous contaminants with documented multifaceted effects across aquatic invertebrates and vertebrates, particularly under chronic low-dose and mixture exposure scenarios (Savoca et al. 2025). Although it is widely accepted that the presence of PAEs in the environment primarily originates from anthropogenic sources, evidence suggests that the biosynthesis of PAEs by different organisms may substantially contribute to environmental contamination (Semenov et al. 2021).

Environmental degradation and fate of PAEs are by abiotic and biotic processes such as hydrolysis, photolysis, and microbial degradation. Bioaccumulation is predominantly by ingestion, but biomagnification is limited (Zhang et al. 2021a; Liao et al. 2023).

The ecotoxicological risk of PAEs is tightly associated with their lipophilicity, high octanol–water partition coefficients (log K_OW_), low vapor pressure, and low volatility, which allow for their diffusion into water and bioaccumulation in biota tissues (Zhang et al. 2021a).

In general, different studies have supported the persistent occurrence of PAEs such as DBP and DEHP in water bodies, even sometimes beyond ecological safety levels (Sun et al. 2021).

Despite stronger regulations, prohibitions and restrictions on certain PAEs in most countries, coping with the global occurrence and environmental impact of PAEs is still a great challenge. This is not only due to their widespread use and recalcitrant character but also due to their complex and largely under-investigated toxicological effects on aquatic species. Clarification of the complex issues is crucial to encourage ecological risk assessment and novel mitigation strategies such as bioremediation in algae and toxicological impact studies in fish. PAEs exhibit multiple toxicity for a variety of organisms. Their dose-dependent toxicity is influenced by their type, duration of exposure, environmental conditions, and species-specific sensitivities (Zhang et al. 2021a; Savoca et al. 2023).

Importantly, fish represent a major dietary pathway for human exposure to PAEs and their metabolites. Therefore, the documented endocrine, developmental, metabolic and epigenetic disruptions observed in aquatic organisms raise concerns for food safety and public health (Meeker et al. 2009) . Alterations in steroidogenesis, thyroid signalling, neurodevelopment and metabolic homeostasis in fish mirror pathways implicated in human reproductive disorders, metabolic syndrome and neurodevelopmental impairment (Dong et al. 2019; Hlisníková et al. 2020). Consequently, aquatic toxicological evidence provides an early-warning system for potential human health risks: as endocrine-active substances (EAS), PAEs interfere with hormonal processes and, depending on the exposure situation, are engaged as risk factors for multifactorial diseases such as reproductive and respiratory disorders, embryogenic malformations and metabolic syndromes (Pham et al. 2025).

This review aims to provide a detailed synthesis of current knowledge on the impacts of PAEs on two key aquatic taxa, algae and fishes, focusing on exposure effects and species-specific sensitivities. It explores the multifaceted role of algae as bioindicators, bioremediators, and potential sources of phthalate pollution. In fishes, it addresses a range of toxicological outcomes, including developmental, reproductive, cardiac, epigenetic, and metabolic effects. By concentrating on these two organism groups, this review offers an integrated perspective on the biological responses and ecological implications of PAEs in marine and freshwater aquatic ecosystems.

In natural aquatic environments, PAEs rarely occur in isolation but interact with co-occurring stressors such as warming, hypoxia, pharmaceuticals and microplastics, leading to non-linear and often unpredictable biological responses (Boahen et al. 2025). Recent studies demonstrate that these combined pressures can amplify endocrine disruption, oxidative stress and metabolic dysfunction in aquatic organisms (Cojoc et al. 2024; Martino et al. 2025), highlighting the limitations of single-contaminant assessments. Unlike previous reviews that primarily address the occurrence or single-species toxicity of PAEs, this work provides an integrated synthesis of PAEs effects across two key aquatic trophic levels: primary producers (algae) and vertebrate consumers (fishes). The novelty of this review lies in (i) jointly analysing algal and fish responses to PAEs, highlighting cross-trophic vulnerabilities; (ii) emphasizing species- and life-stage-specific sensitivities under environmentally relevant exposure scenarios, including mixture effects and metabolites; and (iii) discussing the dual ecological role of algae as both targets and potential sources of phthalates, while simultaneously evaluating their emerging application in bioremediation strategies. By bridging ecotoxicology, physiology and ecosystem functioning, this review moves beyond descriptive toxicity and offers a mechanistic and ecological framework that supports risk assessment, remediation development and regulatory decision-making.

## PAEs on algae

Algae are foundational components of aquatic ecosystems, acting as primary producers and key determinants of ecological balance (Stevenson 2014). They play a central role in aquatic ecosystems by supporting biogeochemical cycles and food webs, but may also threaten ecosystem services when toxic or invasive species proliferate. Therefore, algae are used both as indicators of contamination and habitat alterations, and as proxies for the status of ecosystem goods and services.

Based on size, algae are classified into the following two categories: microalgae and macroalgae (Singh and Saxena 2015). Microalgae have attracted considerable scientific interest due to their short life cycles, high surface-to-volume ratio, and sensitivity to environmental toxicants, making them reliable bioindicators for assessing the impacts of toxic substances on aquatic structure and function (Wenchao et al. 2023). In addition, microalgae represent a valuable source of bioactive compounds and offer a wide range of potential biotechnological applications, including industrial and municipal wastewater remediation (Jalilian et al. 2020).

Although comparatively less studied, macroalgae are also increasingly employed in biomonitoring and bioremediation applications, particularly due to their capacity to bioaccumulate pollutants (García-Seoane et al. 2018; Raj et al. 2022).

However, both microalgae and macroalgae may also contribute to aquatic pollution through the release of secondary metabolites, some of which can exert toxic effects on aquatic organisms (Ethiraj and Samuel 2024; Martino et al. 2024). Among these compounds are PAEs, which have long been considered pollutants of exclusively anthropogenic origin (Pace et al. 2024; Enikeev 2025). In fact, recent studies suggest that PAEs are not merely exogenous contaminants but are also endogenously synthesized by different organisms including algal species as secondary metabolites in response to stressful conditions, where they can act as chemical defences against microbial competitors and modulators of microbial community population dynamics (Pace et al. 2024). It was demonstrated that both freshwater algae and cyanobacteria have the capacity to produce monoethylhexyl phthalate (MEHP) or dibutyl phthalate (DBP) under natural conditions, which can then be released to aquatic environments (Baloyi et al. 2021). Notably, natural PAEs can be distinguished chemically from petrochemical analogues based on their greater content of ^14^C (Chen 2004).

This dual role positions algae both as susceptible targets of phthalate toxicity and potential sources of their natural environmental presence, reflecting their broader capacity to produce bioactive secondary metabolites (Badalamenti et al. 2026).

At the same time, algae also exhibit substantial potential as bioremediators of PAEs via multiple mechanisms including bioadsorption, intracellular bioaccumulation, and enzymatic degradation (Kumar et al. 2023). For instance, in a 31-day exposure experiment, *Ulva lactuca* thalli were exposed to sediments contaminated with a mixture of six phthalates: dimethyl phthalate (DMP), diethyl phthalate (DEP), dibutyl phthalate (DBP), benzyl butyl phthalate (BBP), di(2-ethylhexyl) phthalate (DEHP), di-n-octyl phthalate (DnOP). The results revealed marked compound bioaccumulation, highlighting the suitability of this macroalga for bioremediation applications (Savoca et al. 2022).

Bioremediation efficiency varies widely among algal species and is phthalate-type, concentration, and surrounding environmental parameter dependent. In particular, some algae can metabolize PAEs into less toxic or more hydrophilic compounds, which promotes their further removal or detoxification in aquatic environments.

Significantly, the presence of phthalates as mixtures that occur in real-world aquatic environments can result in synergistic or antagonistic interactions that alter both the toxicity pattern and the degradation kinetics. These interactions can affect algal metabolic pathways, inhibit photosynthetic activity, and alter phthalate uptake and transformation kinetics, thereby regulating the overall bioremediation efficiency and ecological risk assessment.

For instance, Chi et al. (2019) investigated the interactive effects of DEP and DBP on the growth and chlorophyll of three marine microalgae, *Chaetoceros muelleri*, *Cylindrotheca closterium*, and *Dunaliella salina*, and documented species-specific responses to phthalate. Tolerance was compound-specific: for DEP, it was *C. muelleri* > *C. closterium* > *D. salina*, whereas for DBP, it was *C. closterium* > *C. muelleri* > *D. salina*. In general, the most sensitive green alga was *D. salina*, whereas the benthic diatom *C. closterium* had a greater adaptive capacity. Moreover, variability of PAEs degradation efficiencies among the microalgal species examined was related to the cell surface area-to-volume (S/V) ratio. The S/V ratio of the benthic alga *C. closterium* was less than that of the two planktonic species. Therefore, some morphological features and physicochemical surface properties of *C. closterium* cells might facilitate intracellular degradation of DBP. Following surface association, PAEs undergo light-driven photodegradation under natural sunlight. This process involves photoinduced decarboxylation, hydroxylation, dealkylation, and the cleavage of C–O, C–C and O-alkyl bonds, contributing to their transformation into less complex or more biodegradable intermediates (Amirian et al. 2025).

Importantly, Chi et al. (2019) determined that bioconcentration and biodegradation were both species-dependent and influenced by the chemical nature and concentration of the pollutants. During individual testing of DEP and DBP, microalgae were more efficient at biodegradation, pointing to active involvement of algal metabolism in PAE degradation. However, when co-exposed, the two PAEs reduced the overall biodegradation potential, which caused higher bioconcentration of these contaminants in algal cells. This outcome indicates potential antagonistic effects under combined contaminant scenarios.

Apart from the large database on acute and chronic aquatic toxicity published by Staples et al. (1997), recent publications still limited insight on the sublethal and long-term action of PAEs on marine algae, highlighting the need for further ecotoxicological studies in this area. Table 1 summarizes reported effective dose and the lethal dose in different species of marine and freshwater microalgae.

**Table 1:** Summary of effects observed in microalgae

Organism	Environment	Toxicity descriptors/exposure time—**PAEs**: concentration values	Test endpoint/criteria—effect observed	references
*Karenia brevis* (Davis) Gert Hansen & Moestrup, 2000 (Dinoflagellata)	Seawater	96 h-EC_50_—**DBP**: 13.9 mg L^−1^	Inhibitory effects and oxidative stress	Liu et al. 2016
*Phaeodactylum tricornutum *Bohlin, 1897 (Ochrophyta)	Seawater	96-h EC_50_—**DMP**: 390 mg L^−1^ 96-h EC_50_—**DEP**: 74 mg L^−1^	Growth inhibition	Gao et al. 2021
*Chaetoceros muelleri* Lemmermann, 1898 (Ochrophyta)	Seawater	48 h-EC_50_—**DEP**: 160 mg L^−1^ 72 h-EC_50_—**DEP**: 169 mg L^−1^ 96 h-EC_50_—**DEP**: 194 mg L^−1^ 48 h-EC_50_—**DBP**: 3.5 mg L^−1^ 72 h-EC_50_—**DBP**: 3.5 mg L^−1^ 96 h-EC_50_—**DBP**: 3.4 mg L^−1^	Growth inhibition	Chi et al. 2019
*Cylindrotheca closterium* (Ehrenberg) Reimann & J.C. Lewin, 1964 (Ochrophyta)	Seawater	48 h-EC_50_—**DEP**: 52 mg L^−1^ 72 h-EC_50_—**DEP**: 67 mg L^−1^ 96 h-EC_50_—**DEP**: 77 mg L^−1^ 48 h-EC_50_—**DBP**: 3.0 mg L^−1^ 72 h-EC_50_—**DBP**: 3.0 mg L^−1^ 96 h-EC_50_—**DBP**: 4.2 mg L^−1^	Growth inhibition	Chi et al. 2019
*Dunaliella salina* Teodoresco, 1905 (Chlorophyta)	Seawater	48 h-EC_50_—**DEP**: 71 mg L^−1^ 72 h-EC_50_—**DEP**: 65 mg L^−1^ 96 h-EC_50_—**DEP**: 62 mg L^−1^ 48 h-EC_50_—**DBP**: 1.0 mg L^−1^ 72 h-EC_50_—**DBP**: 0.7 mg L^−1^ 96 h-EC_50_—**DBP**: 0.7 mg L^−1^	Growth inhibition	Chi et al. 2019
*Scenedesmus obliquus* (Turpin) Kützing, 1833 (Chlorophyta)	Freshwater	96 h-EC_50_—**DBP**: 15.3 mg L^−1^	Growth inhibition	Gu et al. 2017
*Chlorella pyrenoidosa (Chodat)* Butcher, 1957 (Chlorophyta)	Freshwater	96 h-EC_50_—**DBP**: 3.14 mg L^−1^	Growth inhibition	Gu et al. 2017
*Chlorella vulgaris* Beijerinck, 1890 (Chlorophyta)	Freshwater	LC_50_/EC_50_—**DBP**: 57.6 mg L^−1^ LC_50_/EC_50_—**DEHP**: > 400 mg L^−1^	Death	Yuan et al. 2022
*Chlorella vulgaris* Beijerinck, 1890 (Chlorophyta)	Freshwater	24-h EC_50_—**DEP**: 3539.44 mg L^−1^ 48-h EC_50_—**DEP**: 244.211 mg L^−1^ 72-h EC_50_—**DEP**: 144.486 mg L^−1^ 96-h EC_50_—**DEP**: 122.804 mg L^−1^	Inhibitory effects	Liang et al. 2024

Importantly, most toxicity values reported for microalgae, including those for *Chlorella*, *Scenedesmus*, *Dunaliella*, *Chaetoceros*, and *Cylindrotheca* species, fall within the mg L^−1^ range (Table 1), far exceeding concentrations commonly measured in surface waters, which are typically in the ng L^−1^ to low μg L^−1^ range. At these environmentally relevant levels, several studies report no significant inhibition of algal growth, as exposure concentrations remain below NOEC thresholds (e.g. Chi et al. 2019). Accordingly, existing laboratory studies predominantly describe acute growth inhibition, oxidative stress responses, and cellular damage under controlled high-dose exposures (e.g.Chi et al. 2019; Liang et al. 2024 Gu) and should therefore be interpreted primarily as indicators of algal sensitivity and hazard thresholds rather than direct predictors of environmental risk. At present, data addressing sublethal or chronic effects of PAEs on algae at environmentally relevant concentrations remain scarce. Nevertheless, given the high surface-to-volume ratio of microalgae and their central role at the base of aquatic food webs, even subtle metabolic or photosynthetic perturbations at low µg L^−1^ levels could propagate through trophic interactions. This knowledge gap highlights the need for future studies focusing on long-term, low-dose exposures, mixture toxicity, and environmentally realistic exposure regimes to better integrate algal responses into ecological risk assessment frameworks.

The toxicity of PAEs to microalgae is influenced both by the chemical structure of the compounds and by the species exposed. In general, monoesters are less toxic than diesters. For example, Jonsson and Baun (2003) compared the toxicity of some monoesters (mono-methyl phthalate (MMP), mono-ethyl phthalate (MEP), mono-butyl phthalate (MBP), mono-benzyl phthalate (MbenzP), mono-(2-ethylhexyl) phthalate (MEHP), mono-decyl phthalate (MdecP)) and diesters (DMP, DEP, DBP, BBP, DEHP), including o-phthalic acid, to the green alga *Pseudokirchneriella subcapitata*. Their results clearly demonstrated a lower toxicity level of monoesters compared to diesters.

The sensitivity towards a specific phthalate can also vary significantly among algal species. As reported by Gu et al. (2017), exposure to DBP (2–20 mg L^−1^) strongly inhibited the growth of both *Scenedesmus obliquus* and *Chlorella pyrenoidosa*. The 96-h median effective concentration (96 h-EC_50_) values were 15.3 mg L^−1^ for *S. obliquus* and 3.14 mg L^−1^ for *C. pyrenoidosa*, indicating a higher sensitivity of the latter to DBP. In contrast, Yuan et al. (2022) found *Chlorella vulgaris* to be comparatively insensitive to DEHP and DBP, suggesting species-specific tolerance mechanisms.

Inhibition of algal growth by phthalate exposure is also accompanied by reduced cell density and chlorophyll content, maybe due to oxidative stress mechanisms. Gu et al. (2017) noted intracellular reactive oxygen species (ROS) accumulation and malondialdehyde (MDA) levels, which cause lipid peroxidation and damage to cell integrity.

Beyond these general stress responses, molecular-level evidence indicates that phthalates can directly interfere with specific metabolic and signalling pathways in microalgae. Liao et al. (2020) investigated the effects of di-*n*-butyl phthalate (DBP) on *Chlorella vulgaris* using a combined proteomic and gene ontology (GO) approach. Acute toxicity assays revealed a 24 h EC_50_ of 4.95 mg L^−1^, associated with reduced chlorophyll *a* (Chl-*a*) content and intracellular accumulation of DBP. Proteomic analysis identified 1257 proteins, with DBP exposure inducing a general upregulation of protein expression. Notably, key enzymes involved in primary metabolism, including acetolactate synthase (ALS) and GDP-*L*-fucose synthase 2 (GER2), were downregulated by more than 1.5-fold. Functional annotation indicated inhibition of the valine biosynthetic pathway and nucleotide-sugar metabolism, suggesting that DBP disrupts amino acid biosynthesis and cell wall–related carbohydrate processes critical for algal growth and cellular integrity.

More recently, pathway-oriented studies have expanded this mechanistic framework by linking phthalate exposure to redox imbalance, mitochondrial dysfunction, and programmed cell death (PCD) in microalgae. In *Chlorella vulgaris*, dimethyl phthalate (DMP) exposure (0, 100, 200, 500 mg L^−1^) triggered excessive intracellular reactive oxygen species (ROS) accumulation, lipid peroxidation, and loss of membrane integrity, accompanied by biphasic modulation of antioxidant enzymes (superoxide dismutase, SOD; catalase, CAT) and depolarization of the mitochondrial membrane potential (MMP) (Li et al. 2025). Notably, under blue-light illumination, low DMP concentrations (100 mg L^−1^) exerted negligible effects on algal growth, whereas high DMP loads (500 mg L^−1^) caused pronounced growth inhibition (≈59%), indicating a dose-dependent threshold in algal tolerance. At elevated concentrations, redox imbalance induced compensatory increases in antioxidant defences, including enhanced carotenoid accumulation and upregulation of SOD and CAT; however, these responses were insufficient to prevent membrane damage and mitochondrial dysfunction.

These effects culminated in the activation of caspase-dependent apoptotic pathways, predominantly through the intrinsic mitochondrial signalling cascade (Cyt *c*–Apaf-1–Caspase-9/3), highlighting a direct link between oxidative stress, impairment of the electron transport chain (ETC), and cell fate regulation. In contrast, at lower DMP levels, blue light promoted photoreceptor-mediated metabolic reprogramming that enhanced carotenoid biosynthesis and ROS scavenging, thereby preserving mitochondrial function and suppressing apoptosis initiation. Proteomic, gene ontology (GO), and KEGG pathway enrichment analyses further revealed a coordinated metabolic shift characterized by the upregulation of energy-generating pathways (tricarboxylic acid cycle, TCA; oxidative phosphorylation), fatty acid (FA) biosynthesis, and isoprenoid metabolism via the methylerythritol phosphate (MEP) pathway, alongside the downregulation of nitrogen assimilation and non-essential growth-related processes (Li et al. 2025).

Complementary evidence in marine microalgae supports a conserved mechanistic link between phthalate exposure, oxidative imbalance, and cellular energy disruption. In *Isochrysis zhanjiangensis*, di-(2-ethylhexyl) phthalate (DEHP) induced concentration- and time-dependent cytotoxicity, characterized by increased malondialdehyde (MDA) levels, glutathione (GSH) depletion, reduced chlorophyll *a* content, and suppression of key antioxidant enzymes, including SOD and glutathione peroxidase (GPx) (Liu et al. 2025). Notably, DEHP exposure significantly decreased lactate dehydrogenase (LDH) and ATPase activities, indicating impairment of glycolytic flux, ATP turnover, and overall cellular energy metabolism. Collectively, these findings demonstrate that phthalate toxicity in microalgae extends well beyond nonspecific growth inhibition, involving coordinated disruption of redox homeostasis, biosynthetic pathways, mitochondrial function, and energy-producing processes.

Wenchao et al. (2023) employed a dielectric spectroscopy-based method to ascertain the impact of increasing concentrations of DEP on *C. pyrenoidosa*. The results indicated the inhibition of algae growth to a great extent, with cell density and biomass decreasing by up to 11.92 × 10^4^ cells mL^−1^ and 19.19 mg L^−1^ at 50 mg L^−1^ DEP, respectively.

Liang et al. (2024) also demonstrated that DEP imposes complex toxicities on *Chlorella vulgaris*, including significant growth inhibition (10.3%–83.47%), ultrastructural disruption of chloroplasts, and significant reduction of photosynthetic pigments (8.95%–73.27%). Metabolic activity, mitochondrial activity and membrane integrity were also disrupted under DEP exposure, ultimately inducing apoptosis in a concentration-dependent manner. These were mechanistically explained by disrupted calcium homeostasis and the upregulation of apoptosis-related genes such as Caspase-3, Caspase-8, and Caspase-9. Transcriptomic analysis revealed that DEP interfered with key cellular pathways such as photosynthesis, terpenoid biosynthesis, and DNA replication, showing its broad impact on algal physiology.

Despite the growing ecotoxicological literature on the effects of PAEs in freshwater microalgae, studies investigating their impact on marine microalgal species remain scarce.

Among the few available studies, Li et al. (2015) investigated the effect of DBP on the marine dinoflagellate *Karenia brevis* and reported that oxidative stress was induced at concentrations as low as 5 mg L^−1^. This was followed by damage to the cell membrane, vacuole structure and inhibition of the activity of mitochondrial superoxide dismutase (Mn-SOD) enzyme. In agreement with these findings, Liu et al. (2016) demonstrated that various PAEs, including DEP, di-allyl phthalate (DAP), di-n-butyl phthalate (DnBP), di-isobutyl phthalate (DiBP), and BBP, elicited a dose-dependent inhibition of *K. brevis* growth, accompanied by increased lipid peroxidation and structural malformations of algal cells. The study identified mitochondria and chloroplasts as key sites of reactive oxygen species (ROS) production, thereby linking oxidative stress to the disruption of essential metabolic pathways and cellular integrity (Liu et al. 2016). Notably, the results revealed that different phthalates may induce comparable oxidative responses through distinct subcellular targets; for example, DEP predominantly stimulated ROS production in mitochondria, DAP and DBP affected both mitochondria and chloroplasts, while BBP induced ROS generation mainly in chloroplasts of *K. brevis* cells (Liu et al. 2016). These results collectively highlight the vulnerability of marine microalgae to phthalate-induced oxidative damage, underscoring the need for integrated ecotoxicological risk assessments in marine ecosystems.

Unlike vertebrate taxa such as fishes, for which detailed molecular, endocrine, and signalling pathways affected by phthalate exposure have been extensively characterized, mechanistic studies in algae remain comparatively scarce. Future research should therefore prioritize the elucidation of phthalate-induced alterations in specific algal pathways, including transcriptomic, proteomic, and epigenetic responses, to better integrate primary producers into adverse outcome pathway (AOP) frameworks.

## PAEs on fishes

PAEs represent one of the most widely investigated classes of endocrine-disrupting chemicals in aquatic organisms, with numerous studies focusing on fish models. This is due to the fact that they were highly employed as plasticisers and consistently present in water bodies, thereby being a priority for biomonitoring programmes (Net et al. 2015). PAEs were used in fish to evaluate endocrine disruption, reproductive toxicity, and developmental toxicity due to the availability of well-established test species and adequate understanding of the endocrine regulation of the species. Moreover, fish are established toxicological models that have well-characterized endpoints for acute and chronic toxicity (Pham et al. 2025). This facilitates hazard identification, ecological risk assessment, and regulatory decision-making. The following table (Table 2) gives an overview of recent studies on phthalate exposure in different aquatic habitats and their impacts.

**Table 2:** Summary of effects observed in fish

Organism	Environment	Toxicity descriptors/exposure time—**PAEs**: concentration values	Test endpoint/criteria—effect observed	References
*Danio rerio* (Hamilton, 1822)	Freshwater	**DBP**: 0.1 mg L^−1^, 0.5 mg L^−1^	Synergistic endocrine disruption	Chen et al. 2014a
	Freshwater	14 d—**DEP**: 0.08–10 mg L^−1^ 14 d—**BBP**: 0.02–2.5 mg L^−1^ 14 d—**DiBP**: 0.008–0.1 mg L^−1^	Steroidogenesis disruption	Sohn et al. 2016
	Freshwater (embryos)	24–96 hpf—**DEHP**: 200 µg L^−1^, 400 µg L^−1^	Thyroid axis activation	Jia et al. 2016
	Freshwater (embryos)	0–72 hpf—**BBP**: 0.6 mg L^−1^, 1.2 mg L^−1^	Developmental and cardiac malformations	Sun and Liu 2017
	Freshwater (embryos)	0–72 hpf—**DBP**: 1.8 µM, 3.6 µM	Developmental and cardiac toxicity	Sun and Li 2019
	Freshwater	Chronic—**DiNP**: 0.42, 4.2, 42, 420, 4200 mg L^−1^	Reproductive impairment	Santangeli et al. 2017
	Freshwater	30 d—**DBP**: 1133 µg L^−1^ 30 d—**DiBP**: 1038 µg L^−1^	Reproductive hormone imbalance	Chen et al. 2019
	Freshwater (embryos)	0–72 hpf—**DBP**: 50 mg L^−1^	Proteomic alterations	Dong et al. 2018
	Freshwater	60 d—**DEHP**: 100 µg L^−1^	Metabolic and microbiome disruption	Buerger et al. 2019; 2020
	Freshwater (embryos)	24–168 hpf—**MEHP**: 4—500 µg L^−1^	Pancreatic malformations and oxidative stress	Jacobs et al. 2018
	Freshwater (embryos)	0–5 dpf—**MEHP**: 50 µg L^−1^ (F₀); F₁–F₂ unexposed	Transgenerational epigenetic changes	Kamstra et al. 2017
	Freshwater	Chronic—**DEHP**: 0.02–100 mg L^−1^	Gonadal epigenetic reprogramming	Ma et al. 2018
	Freshwater (embryos)	0–72 hpf—**DEHP**: 2.5 mg L^−1^	Wnt/β-catenin–mediated developmental toxicity	Üstündağ et al. 2017
	Freshwater—in vitro	In vitro 4 d—**DEHP**: 0.5, 5, 10 mg L^−1^ In vivo 4 d—**DEHP**: 10 mg L^−1^	ER stress and insulin resistance	Zhang et al. 2021b
	freshwater (embryos)	0–96 hpf—LC_50_—**DEHP**: 54 mg L^−1^; sublethal 10 mg L^−1^	General toxicity and genotoxicity	Boran and Terzi 2019
	Freshwater (embryos)	Parental chronic—**DBP**: 11, 113, 1133 µg L^−1^ Parental chronic—**DiBP**: 10, 103, 1038 µg L^−1^ F₁ 0–96 hpf same doses	Reproductive and offspring effects	Chen et al. 2021
	Freshwater (embryos)	4 months parental—**DBP**: 4.9, 13.6, 43.8 µg L^−1^; F₁ monitored to 5 dpf	HPG-axis–mediated reproductive toxicity	Hu et al. 2023
	Freshwater (embryos)	0–96 hpf—**DBP**: 50 mg L^−1^, 250 mg L^−1^ 0–96 hpf—**DEHP**: 50 mg L^−1^, 250 mg L^−1^	Cardiotoxicity and epigenetic remodelling	Mu et al. 2020
	Freshwater	21 d—**MEHP**: 2, 10, 50 mg L^−1^	Female-specific endocrine disruption	Park et al. 2020
	Freshwater	15 d—diheptyl phthalate **(DHpP)**: 50, 100 mg L^−1^ 15 d—di-isodecyl phthalate **(DiDP)**: 50, 100 mg L^−1^	Neurotoxicity and oxidative stress	Poopal et al. 2020
	Freshwater (embryos)	7 d—**DMP**: 0.0005–250 mg L^−1^ 7 d—**DEP**: 0.0005–250 mg L^−1^ 7 d—**DBP**: 0.0005–250 mg L^−1^ 7 d—**BBP**: 0.0005–250 mg L^−1^ 7 d—**DEHP**: 0.0005–250 mg L^−1^ 7 d—**DnOP**: 0.0005–250 mg L^−1^	Developmental and skeletal toxicity	Pu et al. 2020
	Freshwater (embryos)	24–144 hpf—**DBP**: 50—250 mg L^−1^ 24–144 hpf—**DEHP**: 50—250 mg L^−1^	Neuro-musculoskeletal development	Qian et al. 2020
	Freshwater—in vitro	In vitro 0.5–1 h—**DEHP**: 1 µM In vivo 12 h—**DEHP**: 1 µM	Testis and liver toxicity	Batista-Silva et al. 2020
	Freshwater (embryos)	2–120 hpf—**DMP**: 0–500 mg L^−1^ 2–120 hpf—**DEP**: 0–500 mg L^−1^ 2–120 hpf—**BBP**: 0–500 mg L^−1^ 2–120 hpf—**DnOP**: 0–500 mg L^−1^ 2–120 hpf—**DEHP**: 0–500 mg L^−1^ 2–120 hpf—di-isononyl phthalate **(DiNP)**: 0–500 mg L^−1^	Neurodevelopmental and growth effects	Tran et al. 2021
	Freshwater	5 d—**DEP**: 8.06, 16.12 µM	Endocrine and developmental toxicity	Tseng et al. 2021
	Freshwater	96 h LC_50_—**DMP**: 45.8 mg L^−1^	Death	Cong et al. 2020
	Freshwater (juveniles)	96 h LC_50_—**MEHP**: 0.742 mg L^−1^	Death	Wu et al. 2025
	Freshwater	24 h LC_50_—di-propyl phthalate **(DPRP)**: 16.79 mg L^−1^ 48 h LC_50_—**DPRP**: 9.99 mg L^−1^ 72 h LC_50_—**DPRP**: 6.14 mg L^−1^ 96 h LC_50_—**DPRP**: 3.96 mg L^−1^ 120 h LC_50_—**DPRP**: 1.19 mg L^−1^ 144 h LC_50_—**DPRP**: 0.92 mg L^−1^	Death	Shen et al. 2025
	Freshwater	72 hpf—**DBP**: 2 μg L^−1^	Neuroendocrine and behavioural effects	Tao et al. 2025
	Freshwater (embryos)	6–96 hpf—**DPRP**: 1, 2, 4 mg L^−1^	Embryonic and developmental toxicity	Shen et al. 2025
	Freshwater (embryos)	24–96 hpf LC_50_—**DEP**: 8.408 mg L^−1^ sub‑LC_35_ 3.125, 6.25 mg L^−1^ 24—96 hpf LC_50_—**BBP**: 0.563 mg L^−1^; sub‑LC_35_ 0.125, 0.25, 0.5 mg L^−1^	Neurobehavioural effects	Kim et al. 2024
*Pseudobagrus fulvidraco* (Richardson, 1846)	Freshwater	8 wk chronic—**DEHP**: 0, 0.1, 0.5 mg L^−1^	Lipid metabolism alterations	Meng et al. 2018
*Oreochromis niloticus* (Linnaeus, 1758*)*	Freshwater	7 d—**DEHP**: 50 µg L^−1^	Transcriptomic effects (liver)	Zhang et al. 2018
		24–96 h—**DBP**: 10 mg L^−1^	Genotoxicity in erythrocytes	Benli et al. 2016
		96 h LC_50_—**DBP**: 11.8 mg L^−1^	Death	Benli et al. 2016
*Pimephales promelas* (Rafinesque, 1820)	Freshwater (embryos)	48 h embryo, 14 d larval—**DEHP**: ≤ 0.003 mg L^−1^	Epigenetic effects	Wood et al. 2016
*Cyprinus carpio* (Linnaeus, 1758)	Freshwater	96 h LC_50_—**DBP**: 35 mg L^−1^ 96 h LC_50_—**DEP**: 53 mg L^−1^ 35 d sublethal for **DBP**/**DEP**	Behavioural, haematological, and biochemical toxicity	Poopal et al. 2017
*Labeo catla* (Hamilton, 1822)	Freshwater	96 h LC_50_—**DEP**: 16.2 mg L^−1^	Death	Ganguly et al. 2025
		30 d—**DEP**: 0.32, 1.62 mg L^−1^	Endocrine disruption and organ toxicity	
*Labeo rohita* (Hamilton, 1822)	Freshwater	96 h LC_50_—**DEP**: 4.38 mg L^−1^	Death	Latif 2020
*Channa striatus* (Bloch, 1793)	Freshwater	96 h LC_50_—**DEP**: 70 mg L^−1^	Death	George et al. 2017
*Oryzias latipes* (Temminck & Schlegel, 1846)	Freshwater	7–28 dpf—**DEHP**: 20, 100, 200 µg L^−1^	Developmental toxicity and oxidative stress	Yang et al. 2018
*Oryzias melastigma* (McClelland, 1839)	Seawater	1–10 dpf—**DEHP**: 0.01, 0.1, 1 mg L^−1^	Accumulation and endocrine disruption	Ye et al. 2014
*Dicentrarchus labrax* (Linnaeus, 1758)	Seawater—in vitro	24 h—**DEHP**: 0.01, 0.02, 0.1, 0.2, 0.5, 1, 2, 5 mM	Cytotoxicity and genotoxicity	Molino et al. 2019
*Sparus aurata* (Linnaeus, 1758)	Seawater	21 d—**DiNP** in feed	Metabolic and neuroendocrine effects	Forner-Piquer et al. 2018

Fish and *Danio rerio* in particular have developed as crucial models for evaluating the toxicological impact of parent PAEs and their metabolites because they are highly fertile, have transparent embryos, mature sexually quickly, are small in size, and are cheaply maintained (Bailey et al. 2013; Dai et al. 2014).

Several studies have identified that co-occurring PAEs and synthetic oestrogens synergistically augment tissue-level toxicity: for instance, Chen et al. (2014a) demonstrated that while long-term exposure (20–115 days post-fertilization, dpf) of zebrafish to DBP (0.1 and 0.5 mg L^−1^) individually did not impact growth, induction of vitellogenin, or sex ratio, confirming DBP’s low oestrogenic activity, binary mixtures of EE2 (17*α*‑ethinylestradiol; 5 and 20 ng L^−1^) produced EE2-dominant impacts on endocrine endpoints but increased histopathological damage (cellular swelling, nuclear atrophy, vacuolization, epithelial hypertrophy, oedema and telangiectasis) in gonad, liver and gill beyond that induced by either compound individually. Moreover, the fact that sex ratios in mixtures mirrored those of EE2 alone indicates that DBP intensifies morphological injury while acting largely independently on endocrine pathways.

Building on these findings, Sohn et al. (2016) exposed male zebrafish to low-molecular-weight PAEs (DEP, BBP, DiBP) and their hydrolytic monoesters, uncovering marked downregulation of star and 3β-hsd genes alongside upregulation of cyp19a (aromatase). As a consequence, testosterone levels declined more steeply than estradiol, yielding an elevated E₂/T ratio, while hepatic vitellogenin expression fell in parallel with reduced estradiol, thereby explaining the observed anti-androgenic phenotype and highlighting that both parent PAEs and their metabolites can severely impair steroidogenesis in fish.

In addition to sex hormone disruption, PAEs interfere with the thyroid axis: Jia et al. (2016) reported that exposure of embryos and larvae to DEHP at 200 and 400 µg L^−1^ upregulated corticotropin-releasing hormone and thyroid-stimulating hormone, increased whole-body thyroxine (T₄) and triiodothyronine (T₃) via elevated thyronine deiodinase activity, and boosted transcription of nkx2.1, thyroglobulin, and transthyretin, while downregulating ugt1ab, collectively indicating activated thyroid hormone synthesis and transport without overt developmental defects at these concentrations.

Furthermore, substantial evidence indicates that PAEs induce dose-dependent developmental and cardiac impairments in zebrafish embryos: Sun and Liu (2017) showed that BBP (0.6 and 1.2 mg L^−1^) elicited yolk-sac oedema, spinal curvature, tail deformity, uninflated swim bladder, reduced hatching and survival rates, abnormal cardiac looping (increased sinus venosus–bulbus arteriosus distance), reduced heart rate, and downregulation of cardiac transcription factors Nkx2.5 and Tbx5 at 72 h post-fertilization (hpf). Similarly, Sun and Li (2019) found that DBP exposure (0–72 hpf) caused pericardial oedema, heart-looping defects, bradycardia, and dose-dependent suppression of NKX2.5 and TBX5 expression. Extending these findings, Mu et al. (2020) directly compared DBP and DEHP exposures (50 and 250 mg L^−1^), revealing that DBP yields stronger cardiotoxicity: both PAEs upregulated cardiac injury markers (nppa, cTnT), induced apoptosis around cardiac tissues, altered expression of tbx5b, myl7, cmlc1, and tmod1, and triggered epigenetic modifications, hypermethylation of tbx5b and hypomethylation of nppa, thus linking DNA methylation changes to defective heart morphogenesis.

More recently, Tao et al. (2025) report that low-dose DBP exposure (2 µg L^−1^) from fertilization to 72 hpf in zebrafish induces not only classic morphogenetic defects, oedema, spinal curvature, reduced survival and hatching, and hyperactive movements with elevated heart rate but also long‑term neuroendocrine dysfunction. Larvae exhibited HPA axis overactivation (reduced CRH, increased ACTH and cortisol), microglia and astrocyte activation, and elevated pro‑inflammatory cytokines, persisting into adulthood with anxiety‑ and depression‑like behaviours, chronic HPA dysregulation and immune pathway disruptions.

Shen et al. (2025) further expand the embryotoxic profile by demonstrating that exposure of zebrafish embryos to dipropyl phthalate (DPRP; 1–4 mg L^−1^, 6–96 hpf) causes dose-dependent mortality, reduced hatching, pericardial oedema, shortened body and craniofacial dimensions, mandibular retraction and cartilage malformations. DPRP impairs locomotion, dysregulates neural crest-derived cartilage gene expression, disrupts pharyngeal arch vascularization, triggers chondrocyte apoptosis/proliferation imbalance, induces inflammation and oxidative stress, and activates FoxO signalling.

Complementing these embryo-centric studies, Kim et al. (2024) evaluated developmental and neurobehavioural effects of DEP and BBP in zebrafish embryos and larvae. Exposures below LC_35_ values (DEP LC_50_ 8.408 mg L^−1^; BBP LC_50_ 0.563 mg L^−1^) did not affect hatching but impaired early motor behaviours (tail coiling, touch-evoked response, light/dark swimming), altered sleep–wake patterns (increased sleep time and bouts; reduced movement and sleep latency for DEP), reduced acetylcholinesterase and dopamine levels (BBP), increased serotonin (BBP), and dysregulated circadian clock gene expression, linking phthalate neurotoxicity to neurotransmitter imbalance and clock disruption.

Moreover, chronic PAE exposures also compromise adult fish reproduction and can engender transgenerational effects. Santangeli et al. (2017) demonstrated that DiNP alters the cellular expression of steroidogenic and folliculogenesis genes (e.g. star, cyp11a, lhcgr, esr, bmp15) in the ovaries, decreases the production of vitellogenic and mature oocytes, and modifies lipid profiles in the follicles. In a related study, Chen et al. (2019) reported that DBP or DiBP individually decreased plasma estradiol, elevated testosterone/estradiol ratios, decreased gonadosomatic indices (GSI), and accelerated early oocyte growth, but co-exposure to combined DnBP + DiBP suppressed such effects, suggesting that some mixture interactions could mitigate some reproductive toxicity characteristics. Proteomics analysis by Dong et al. (2018) further identified DBP-induced upregulation of brain-type creatine kinase and total creatine kinase, RNA-binding proteins (SERBP1, SSB), and receptor-trafficking proteins (SNX1), pointing to impacts on energy homeostasis, developmental stress responses, and neural/muscular systems.

Most strikingly, Kamstra et al. (2017) demonstrated that embryonic MEHP exposure results in heritable and direct DNA methylation alterations at zfCNEs enriched in pathways of adipogenesis (PPARα/γ, TGFβ, WNT7A) and neurodevelopment (SOX2, ASCL1) that set persistent epigenetic marks through two successive generations. Ma et al. (2018) expanded this epigenetic narrative by showing that adult male chronic DEHP exposure leads to cyp17a1 and hsd17b3 promoter hypermethylation, cyp19a1a promoter hypomethylation, reduced sperm counts, reduced fertilization efficiency, and growth impairments in offspring, which put forth the threat of transgenerational endocrine and developmental disruption.

In particular, parental exposure experiments illustrate maternal effects on offspring fitness: Tseng et al. (2021) reported that subacute DEP treatment (8.06–16.12 µM for 5 days) in adult females significantly decreased ovarian estradiol, decreased hepatic vtg1, esr1, and esr2b, and induced reduced hatching rates and craniofacial cartilage deformities in larvae, as well as aberrant swimming behaviour, highlighting pronounced maternal-transfer toxicity mechanisms.

Ganguly et al. (2025) examined chronic diethyl phthalate (DEP) effects in the Indian major carp Labeo catla over 30 days and reported hepatosomatic index increase significantly, activation of HPG axis (upregulation of Kiss1/2, GnRH, FSH, vitellogenin, estradiol, and 11-KT), suppression of antioxidant defence (SOD, CAT) and histopathological damage to liver and kidney and mild inflammatory changes in brain tissue, thereby extrapolating PAE toxicity findings to non-zebrafish species and demonstrating cross-species conservation of endocrine and oxidative stress responses.

In addition to epigenetic and endocrine effects, PAEs induce metabolic deregulation and microbiome impairment: Buerger et al. (2019; 2020) reported DEHP-induced body mass index gain in overfed zebrafish through reprogramming gastrointestinal transcriptional networks (lipid metabolism, PPARα/γ signalling, immune pathways) and gut microbiota diversity compromise with enhanced Bacteroidetes and impaired short‑chain fatty acid production, thus correlating PAE exposure with host–microbiome axis impairment.

Extending the study to other species beyond zebrafish, numerous studies have established PAE toxicity in other species: Ye et al. (2014) exposed marine medaka (*Oryzias melastigma*) embryos to dose-accumulate DEHP (0.01–1 mg L^−1^) without affecting mortality or hatching, but with higher oestrogen-responsive genes (ERα, ERβ, VTG1/2, ChgH/L, CYP19a/b), recovered after depuration; Yang et al. (2018) reported that freshwater medaka (*Oryzias latipes*) larvae (7–28 dph) exposed to 20–200 µg L^−1^ DEHP exhibit reduced growth, lower antioxidant enzymes, alternative PPAR and p53/apoptosis pathways, and neurobehavioural alterations. Additionally, Poopal et al. (2017) documented that DBP and DEP-treated juvenile *Cyprinus carpio* demonstrate osmoregulatory failure, haematological alterations, oxidative stress, and behavioural impairment, while (Benli et al. 2016) noted that juvenile Nile tilapia (*Oreochromis niloticus*) under DBP treatments accumulate genotoxic effects measured in terms of increase in micronuclei frequency. Molino et al. (2019) established cytotoxic and genotoxic activities of DEHP and MEHP, in *Dicentrarchus labrax* embryonal cell lines, and Meng et al. (2018) have reported lipid homeostasis disruption in juvenile yellow catfish (*Pseudobagrus fulvidraco*) after exposures to DEHP for eight weeks.

Moreover, recent studies have highlighted that other metabolic organs are affected by PAEs: Jacobs et al. (2018) revealed MEHP-induced pancreatic malformations and oxidative-stress-mediated endocrine disruption (downregulation of insa, sst2, ptf1a; upregulation of gsr, gstp1), while Zhang et al. (2021b) showed MBP triggers endoplasmic reticulum stress, autophagy, apoptosis, and dysregulation of lipid/glucose metabolism (acc1, fasn, srebp1, PPARα/γ, acox1), resulting in insulin resistance. More recently, Lee et al. (2026) investigated the neurotoxic effects of DBP and its active metabolite MBP in zebrafish (Danio rerio) larvae. Both compounds disrupted early neurodevelopment, reducing neurogenesis (HuC) and myelination (mbp) as well as altering behaviour, including touch-evoked responses and locomotor activity. MBP in particular induced behavioural changes comparable to or exceeding those caused by DBP at maximum non-lethal concentrations, including reductions in dopamine levels and increased oxidative stress, with corresponding activation of antioxidant defences (CuZn-sod, gpx1a, gsta1, gr). These results emphasize that metabolite-mediated toxicity can be as important as, or even more pronounced than, the parent compound, and highlight the need to incorporate metabolites such as MBP into environmental risk assessment and regulatory frameworks.

Recent studies have extended plasticizer toxicity assessment to non-model freshwater fish species. In controlled laboratory experiments, multiple plasticizers (Bisphenol-A, Calcium stearate, and DBP) were tested in *Tilapia sparrmanii* under standardized acute (96 h) and subchronic (21-day) exposure conditions (Mensah et al. 2026). In the 96-h acute test, DBP caused measurable mortality at concentrations of 1–4 mg/L, while lower concentrations did not result in significant lethality. Over the 21-day subchronic exposure, environmentally relevant concentrations of DBP (0.14–0.54 µg L^−1^) elicited significant biochemical alterations, including acetylcholinesterase (AChE) inhibition and increased lipid peroxidation (LPx), indicating disruption of cholinergic neurotransmission and oxidative membrane integrity. These findings highlight that low-dose, sublethal endpoints can reveal biologically meaningful effects relevant to environmental exposures, while the higher-dose tests provide valuable mechanistic insights and enable comparison of toxicity among plasticizers.

In contrast to the algal literature, fish studies increasingly demonstrate that biologically relevant effects occur at concentrations approaching those detected in contaminated aquatic environments. Several investigations report endocrine, developmental, neurobehavioural, and epigenetic alterations in the low µg L^−1^ or sub-µg L^−1^ range, including metabolic and microbiome alterations at 100 µg L^−1^ DEHP (Buerger et al. 2019; 2020), parental and transgenerational effects at tens of µg L^−1^ for DBP and DiBP (Chen et al. 2021; Hu et al. 2023), and neuroendocrine and behavioural dysregulation following embryonic exposure to DBP at 2 µg L^−1^ (Tao et al. 2025). These results suggest that sublethal endpoints targeting endocrine signalling, neurodevelopment, reproduction, and epigenetic programming better capture environmentally realistic toxicity, while higher-dose studies continue to inform hazard identification, mechanistic pathways, and comparative toxicity ranking.

In summary, these studies collectively depict a comprehensive portrait of DBP and other PAE-driven toxicity in fish, ranging from acute lethality at higher concentrations to sublethal biochemical, developmental, endocrine, and epigenetic disturbances at environmentally relevant concentrations, underscoring the complementary value of both exposure ranges for ecological risk assessment.

## General trends in PAE mixture toxicity

At present, most studies on phthalate esters (PAEs) focus on single-compound exposure, particularly DEHP. While these investigations have provided valuable insights into the molecular mechanisms underlying individual PAE toxicity and identified key biological pathways involved in adverse outcomes, organisms in natural aquatic environments are rarely exposed to isolated chemicals. Instead, realistic exposure scenarios involve complex mixtures. Consequently, ecological risk assessment of PAEs requires explicit consideration of mixture toxicity (Liu & Pei 2025).

To predict chemical interactions within mixtures, several modelling approaches are commonly applied, including Concentration Addition (CA), Independent Action (IA), and the Combination Index (CI). The CA model assumes that chemicals share similar mechanisms of action (MoA) and act on the same biological target, resulting in additive effects, and is therefore appropriate for structurally related compounds with comparable toxicity profiles. In contrast, IA is generally applied to chemicals with dissimilar MoA or multiple independent targets. The CI approach allows quantitative evaluation of synergistic or antagonistic deviations across effect levels and concentration ranges (Liu & Pei 2025). Although CA and IA remain widely used reference models, predictive accuracy may decline in the presence of highly potent or biologically interactive mixtures.

Empirical evidence indicates that additive effects generally dominate PAE mixture toxicity, particularly among structurally related short-chain phthalates. Subchronic toxicity tests in green algae (*Raphidocelis subcapitata*), daphnids (*Ceriodaphnia dubia*), and fish (*Danio rerio*) exposed to mixtures of short-chain PAEs (C1–C6) showed good agreement between observed toxicity and CA predictions, with only minor deviations (Oda et al. 2025). These findings support CA as a suitable default model for apical endpoints such as growth inhibition and survival.

However, mixture responses become more complex in vivo, particularly for endocrine-related endpoints. Phthalates such as DEHP, DBP, DnOP, and DEP induce reproductive and developmental toxicity in zebrafish and Oryzias melastigma, reflecting multi-level physiological disruption beyond simple growth inhibition (Chen et al. 2014b; Guo et al. 2016). Binary mixtures, particularly C2–C6 and C11–C15 combinations, have been shown to induce more severe effects than individual compounds, including developmental malformations, increased apoptosis, and marked perturbations of hypothalamic–pituitary–gonadal (HPG) axis genes (erα, erβ, ar, cyp19a, vtg), accompanied by downregulation of cyp19b (Hamid et al. 2020).

In contrast, in vitro assays using microbial and mammalian systems reveal a broader spectrum of interaction patterns: additive, antagonistic, and occasionally synergistic depending on mixture composition, concentration, and endpoint (Hamid et al. 2020). CI- and IA-based comparisons in these simplified systems frequently report antagonistic deviations across a wide effect range (Fa = 0.1–0.9), followed by additive and less commonly synergistic responses (Ding et al. 2017; Hamid et al. 2020). Such antagonism likely reflects competitive binding among structurally similar PAEs at overlapping receptor sites.

Conversely, whole-organism experiments in zebrafish indicate that deviations from IA or CA predictions for endocrine-related endpoints more often manifest as additive or synergistic effects rather than antagonistic ones (Hamid et al. 2020). These findings highlight the capacity of vertebrate endocrine networks, characterized by feedback loops, hormone synthesis, and receptor cross-talk, to amplify mixture responses at organismal levels. Molecular docking analyses support this mechanistic interpretation, demonstrating strong binding affinities of PAEs to ERα and ERβ, with DEHP exhibiting the highest predicted affinity, consistent with its prominent role in driving mixture-related oestrogenic activity (Hamid et al. 2020).

Overall, across taxa and endpoints, the CA model emerges as the most broadly supported and conservative predictive framework for PAE mixtures, particularly for apical toxicity endpoints in algae, invertebrates, and fish. Antagonistic interactions are more frequent in simplified in vitro systems, whereas additive or synergistic responses are more consistently observed for endocrine-mediated in vivo endpoints. Thus, while mixture effects remain species- and endpoint-dependent, CA-based additivity represents the dominant and regulatory-relevant pattern in the current literature.

Importantly, mixture toxicity assessment should also consider co-exposure with other environmental contaminants (e.g. metals, BPA, pharmaceuticals), which can enhance neurotoxic, apoptotic, reproductive, and endocrine-disrupting effects (Hadrup et al. 2013; Hamid et al. 2021). Predictive models such as Generalized Concentration Addition (GCA) are increasingly applied to account for differences in component efficacy, providing more accurate estimations of joint effects in complex environmental mixtures (Watt et al. 2016). Overall, these findings highlight that understanding PAE mixture interactions is essential for accurate environmental risk assessment and informed regulatory decision-making.

## Comparative methodological perspectives in PAE ecotoxicology

Interpretation of PAE ecotoxicity data is strongly conditioned by the experimental approach employed, yet comparative methodological considerations are often underdeveloped. Literature encompasses acute lethality assays, chronic sublethal tests, mechanistic molecular investigations, and mixture modelling frameworks, each providing distinct but non-equivalent levels of ecological insight.

Standardized acute assays (48–96 h LC_50_/EC_50_) remain the most frequently reported endpoints and form the regulatory backbone of hazard classification. However, these tests are commonly performed at concentrations exceeding environmentally measured levels and primarily capture overt apical effects such as mortality or growth inhibition. Consequently, they may underestimate subtle endocrine, developmental, and metabolic perturbations that occur at sublethal exposures.

Chronic and subchronic designs markedly increase sensitivity by evaluating reproductive output, developmental malformations, oxidative stress responses, endocrine biomarkers, and behavioural alterations. Endpoints such as NOEC and LOEC often reveal biologically significant effects in the µg L^−1^ range, highlighting that long-term exposure scenarios provide a more realistic estimation of ecological vulnerability. Nonetheless, interspecies variability and experimental heterogeneity complicate direct cross-study comparisons.

Mechanistic approaches, including gene expression profiling of HPG axis components, thyroid-related genes, mitochondrial markers, and oxidative stress pathways, as well as emerging omics technologies, have substantially advanced understanding of molecular initiating events and adverse outcome pathways. While these tools enhance mechanistic resolution and early-warning capability, they are predominantly laboratory-based and may not fully account for environmental complexity or adaptive responses.

A further limitation of the current literature lies in the predominance of single-compound studies. Environmental exposure occurs as multi-component mixtures, and predictive models such as CA, IA, and GCA are increasingly applied to bridge this gap. Evidence indicates that CA provides a conservative and broadly supported framework for apical endpoints, whereas endocrine-mediated responses frequently exhibit deviations from simplified predictions, particularly in vivo.

Finally, mesocosm and field-based investigations remain scarce, limiting extrapolation from controlled laboratory systems to dynamic natural environments where co-contaminants, trophic interactions, and abiotic factors modulate toxicity.

Taken together, acute assays are indispensable for regulatory screening, but chronic, mechanistic, and mixture-based approaches offer greater ecological realism and sensitivity. A robust risk assessment framework for PAEs therefore requires integration of multiple methodological tiers rather than reliance on single-endpoint toxicity metrics.

## Conclusion

PAEs appear as ubiquitous stressors in aquatic ecosystems and affect primary producers and organisms from higher trophic levels via similar modes of action, including the induction of oxidative stress, endocrine modulation, and changes in metabolic functions. This review contributes new insights into the species- and life-stage-specific vulnerabilities of primary producers and organisms from higher trophic levels under environmentally relevant conditions, e.g. chronic low-dose effects, metabolite toxicity, and the impact of compound mixtures. The review discussion also points out the duality of algae as both a potential target of PAEs’ toxicity and a useful agent in nature-based solutions, although their efficiency is heavily dependent on the species and constrained under mixture toxicity conditions. The existing scientific evidence also suffers from the restriction of applying the single-species approach, short-term studies, and the inadequate examination of chronic low-dose exposure and the potential impact of compound mixture and multiple stressors simultaneously or sequentially. Moreover, cross-trophic mechanisms and the active metabolic role of algae in contaminant dynamics remain poorly resolved. In addition, the limited availability of pathway-resolved molecular data in algae hampers their full integration into adverse outcome pathway frameworks and cross-trophic risk assessment models. Filling these gaps is critical for improving ecological risk assessment as well as effective remediation strategies.

Overall, this integrated perspective can inform more efficacious risk assessment and highlight the necessity of future multi-trophic, long-term, and mechanistically motivated research to inform strategies of mitigation and regulation of aquatic ecosystems.

## Data Availability

The data supporting the findings of this study are available within the paper. Further information generated during the study is available from the corresponding author on reasonable request.
